# Contextually Aided Recovery (CARe): a scientific theory for innate healing

**DOI:** 10.1186/s12998-017-0137-z

**Published:** 2017-02-13

**Authors:** Dave Newell, Lise R. Lothe, Timothy J. L. Raven

**Affiliations:** 1 Anglo European College of Chiropractic, Bournemouth, UK; 2 Kiropraktorene i Grimstad & Lillesand, Grimstad, Norway

**Keywords:** Chiropractic care, Innate, Healing, Contextual effects, Contextual factors, Placebo

## Abstract

**Background:**

The chiropractic profession emerged when scientific explanations for causes of health and disease were still in infancy and the co-existence of notions such as innate healing and vitalism were perhaps admissible within such a historical context. Notwithstanding, within the scientific culture of the 21^st^ Century all healthcare paradigms require evidential support which in regard these early concepts are in large part, absent. Nevertheless, a large body of emerging scientific evidence supports the existence of innate healing phenomena that may explain a plethora of clinical outcomes observed during chiropractic care. However, in contrast to the notion that removing the putative subluxation constitutes the mechanism by which this healing is initiated, the evidentially supported explanation is one that invokes the impact of contextual factors inherent in the skilful care and authority of the healthcare provider. This perspective is presented here as the scientific model of Contextually Aided Recovery (CARe).

**Main body:**

This paper contends that;

1. Contextual effects are powerful and desirable and are triggered by contextual factors present in all therapeutic encounters including those encountered in chiropractic practice.
2. These factors can elicit large clinical effects with substantive evidence supporting pain, immune and motor modulation.
3. The compartmentalisation of specific and non-specific effects is a biologically and scientifically false dichotomy, erroneously invoked to de-legitimise treatment approaches that expertly construct contextual healing scenarios.
4. The use of factors to construct contextual healing scenarios that maximise positive (placebo) and minimize negative (nocebo) effects is a skilful clinical art within the multimodal approach that describes modern chiropractic care and should be presented and defended as a legitimate component of orthodox healthcare

Clinical improvement during chiropractic care, beyond any biologically specific treatment effects of manipulation and other modalities, may be largely understood considering contextual factors as described by a Contextually Aided Recovery (CARe) model.

## Introduction

In 1644 Descartes formulated the idea that the mind and the body are made of distinct and separate substances; extended matter (body) and non-extended matter (mind or soul). This Cartesian dichotomy went on to form the basis of a persistent paradigm where the separation of mind and body became embedded in Western thought [1]. Some historians have seen this split as a necessary solution of freeing science from religious dogma in that scientists were excluded from questions of the soul or mind but could progress with exploration of the biological machine as represented by the body This enabled the wresting of the body from the sanctity and protection of religion and offered it up to science and medicine for investigation [2]. Subsequent to this, the medical paradigm has continued to tacitly characterize the legitimacy of treatment interventions within this framework, exemplified by such terms as psychosomatic, to describe what are seen as somewhat illegitimate complaints due to the dominance of the mind in the symptomatology as compared to the body [3].

Although much progress has been made, illustrated by the rise of the biopsychosocial [4] and patient centered model of care [5], the perception that health and illness are predominantly associated with the body persists as a recent survey of both university students, healthcare workers and the lay public reported [6].

In the last decade or so, mounting evidence has begun to elucidate mechanisms that underlie some of the so called *specific* effects of spinal manipulative therapy as they pertain to improvements in musculoskeletal symptomatology [7–12]. In addition, mechanisms underlying so called *non-specific* effects are increasingly being characterised and not only blur the line between the notion of specific and non-specific but go deep to the entire description of what constitutes legitimate treatment not only within CAM but at the heart of medical practice itself [13]. Recent articles have already outlined this burgeoning area of scientific endeavour and its potential impact within manual therapeutic approaches [14, 15], arising as it does from increasingly detailed neurophysiological mechanisms describing the role of the placebo effect in clinical practice. This article aims to articulate an evidential explanatory framework that presents the imperative of these powerful therapeutic factors. In addition, and in contrast to the historical negativity associated with the language of placebo we also introduce a new descriptive model called Contextually Aided Recovery (CARe).

## The loading of language: from placebo to CARe

Placebo has a bad name and always did. Emerging as it probably did from medieval Europe it referred to the practice of paid mourners who came to ‘*crocodile cry’* at the funeral of someone they didn’t know. Further entrenchment of its charlatan nature over the years led to it being used in general to describe allegations of fraudulent duping of clients most often in medicine with its plethora of potions, pills and snake oils [16]. At the end of the 19^th^ Century, Mesmer [17] became associated with the therapeutic use of magnetism, a newly described scientific phenomena which he used to ‘cure’ various ailments and illnesses. A famous experiment and an early forerunner of the clinical trial had a practicing associate of Mesmer magnetize one of four trees leaving the others untouched. A sickly and blindfolded boy was then asked to approach the trees and decide which were magnetized. Needless to say, given the expectation and tension the boy convulsed at every tree becoming more affected as he distanced himself from the truly magnetized tree. This led to the idea that a mere ‘placebo’ effect generated purely in the head of the patient, was responsible for the patient reported therapeutic effects. Although Mesmer himself fell quickly out of favour, the practice survived for many years providing devotees with healthy businesses ‘curing’ many ills and maladies with the use of magnetism.

In the light of recent advances in understanding, the often-heard dismissal of the legitimacy of some patient complaints, ‘it’s all in your head’ was somewhat nearer to the truth than perpetrators of such Cartesian dualism knew [18]. However, such separation of the influence of the mind on the body with thought, being limited in its ability to directly influence physiology, persisted. Furthermore, there remained much scepticism amongst the medical profession at large who preferred to deal with the physical ailment itself rather than the mind that was attached to it [19].

Placebo then came to represent ‘nothing’, an inert ingredient with no effect and often with fraudulent overtones attached. This idea is correct in that most placebos are indeed inert and do not have an effect generated by the specific ingredient they are made of. For example, it is not the chalk or the sugar in a placebo pill that interacts in some molecular way with pain receptors to generate common and powerful placebo analgesic effects but rather the patient’s own central nervous system (CNS) that generates the analgesia [20]. Here then, the placebo pill or intervention is merely a trigger, wrapped in contextual meaning that initiates an *innate* ability of the CNS to directly modulate ascending nociception. Although placebo analgesia is the most studied phenomenon it appears it is not the only physiological system that the human CNS can modulate via conscious cues. Evidence is now incontrovertible that patient expectation of benefit as constructed by the use of such contextual cues can also powerfully modulate motor and immune function.. For example, in motor function placebo administration can have marked effects on performance [21, 22] and giving placebo antihistamines to patients with dust mite allergy serves to significantly reduce allergic symptoms merely through expectation of an effect on the part of the patient [23]. Similarly, significant modulation of psoriasis can be achieved through the administration of placebo [24], as can supposed functional conditions such as irritable bowel syndrome (IBS) and other gastrointestinal problems, leading some authors to suggest that *“Rather than focusing on a ‘personalized’ choice of drugs based on biomarkers or genes, it might be the doctor–patient communication that needs to be tailored”* [25]. An increasing realization and evidence base that show powerful modulation of previously considered automatic processes is emerging, as generated by the patient given the right context and expectation. These effects are clinically substantive, widespread and not something to be underestimated. Some authors have suggested alternative language to describe this phenomenon to decouple the historically negative semantics of placebo from what are ostensibly desirable effects. For example, Moerman suggested the ‘meaning effect’ [26] while ‘contextual effect’ or ‘contextual healing’ have also been suggested [27]. The contextual effect - i.e., the analgesia, modulated immune or motor response - can be triggered by a raft of contextual factors commonly present in therapeutic encounters. These may include administration of a pill or treatment, powerful words as used by a clinician, the clinical environment itself or the cultural signals engendered by the use of a white coat or the title of “doctor” amongst many others [28]. A recent review included general categories of known factors that support contextual healing; patient-physician relationship (*verbal communication, nonverbal communication*), treatment features (*clear diagnosis, overt therapy and observational learning, patient centred approach, global process of care, therapeutic touch*), and healthcare setting features (*environment, architecture and interior design*) [29]. Contextual factors then are components of the clinical encounter that appear to provide triggers for the contextual effect, i.e., the reduction of symptoms or improvement in the condition. Construction and delivery of contextual factors is not trivial. It requires skill and knowledge on the part of the heath care provider. If carried out incorrectly, contextual factors can cause equally powerful exacerbation of the symptoms or disease process, otherwise known as nocebo [30]. Indeed, the aboriginal tradition of pointing the bone along with social exclusion have been known to be correlated with the actual demise of the individual, and could potentially be seen as an example of extreme nocebo [31]. In this respect the use of CARe as a therapeutic model can be both clinically beneficial if delivered well and harmful if not. In this context therefore it is indistinguishable from descriptions of other legitimate therapeutic interventions.

## The neurophysiology of contextually driven modulation of pain, immune and motor function

### Pain

The characterisation of the neural mechanisms that underlie the contextual modulation of pain in humans has been extensively described [32, 33]. The elements that trigger such effects within manual therapeutic clinical encounters are also emerging [29]. The ability of individuals to innately attenuate the sensation of pain is significant and can be equivalent to up to 8 mg of morphine administered intravenously [34, 35]. These effects have repeatedly been measured in clinical trials designed to determine the analgesic effects of pain relieving pharmaceuticals. Indeed, the placebo arm of such trials is there precisely to measure such effects. In the case of low back pain trials, the effects of several common pharmaceuticals have been shown to be no greater than a placebo [36, 37]. Even in the use of classic opioid drugs, effects are short term, and for chronic low back pain evidence of efficacy is scant [38]. In addition, there is a considerable risk of addiction and harm associated with their use [39, 40]. The fact that the effect of both the placebo and the drug can be very significant compared to no treatment is often not highlighted in the interpretation of such trials where what is highlighted is any substantive difference in effectiveness between the drug and the placebo which is often small or absent. However, if an administration of placebo can reduce pain by statistically and clinically significant amounts in multiple trials in comparison to common analgesic drugs, then why aren’t health care professionals who see patients in pain, not capitalising on the ability of such mechanisms to help manage such pain for their patients, particularly when the use of common analgesic drugs are accompanied by risk of serious side effects [41, 42]. Despite ethical issues surrounding deception [13, 43, 44], including the efficacy of open placebos where the patient knows the intervention is inert [45], the language of clinical trials continues to centre on the elimination of the placebo effect with highlighting any extra effect of the drug or intervention, the *specific effect,* being the central goal. However, where the placebo effect is large and clinically significant the idea of eliminating the benefits of such a phenomenon outside of clinical trials appears nonsensical.

What then is the ingredient that generates the placebo based modulation of pain? Evidence is now overwhelming indicating it to be the patients’ own CNS within which specific identifiable neural pathways are triggered by contextual factors within the clinical encounter. These are real, physical, measurable and clinically significant effects with numerous studies having now comprehensively documented such analgesic effects and the neural pathways that generate them [33, 46–50]. Such studies have shown that contextually modulated analgesia is controlled by a set of anatomically and functionally identifiable neural pathways that involve a range of specific neurotransmitters including endorphins, cholecystokinin, dopamine and anandamide. Generally, these pathways connect paleo-biologically modern cortical processes (underlying conscious awareness of complex social cues) to ancient brain stem modulation of ascending and descending nociceptive spinal pathways via connections with emotional and reward systems [51–53]. This modulation can both enhance (nocebo) and attenuate (placebo) pain, based on the meaning to the patient of contextual factors present within a clinical encounter. Present knowledge suggests the interpretation of the meaning of these factors in the context of the patient's understanding appear to operate through psychological mechanisms such as expectation and classical conditioning as do the other marked effects outside of pain modulation documented below. In short, how a patient understands and interprets the words and actions of a clinician and the clinical environment within a clinical encounter can switch on or off neurobiological pathways that directly reduce or enhance pain.

### Immune function

The impact of CNS modulation on immune function is not as well characterised as that describing analgesia. However, studies are increasingly documenting the effects of both expectation and classical conditioning as triggered by contextual factors in clinical encounters across a range of immune based health conditions. This emerging evidence is beginning to illuminate the mechanisms involved in connecting immune function with higher cognitive processes. For example, studies have shown that placebo antihistamine can markedly reduce symptoms in a range of allergies if administered under conditions that elicit expectation of effect as signalled by clinical context [54]. These effects do not seem to be limited to self-reported symptoms in that cellular changes in immune cell counts and activity can be measured if classical conditioning is involved (i.e., pairing an inert signal such as novel tasting drink with immunosuppressive drug) [55]. Indeed, such conditioning has been suggested as a new therapeutic approach in reducing immunosuppressive drug dose by harnessing the innate ability of neural-immune system communication to learn an effective placebo [56].

Like placebo induced analgesia, this evidence suggests that conscious interpretation of context, and the meaning this has for the patient, can be used by the CNS to actively modulate immune processes down to the cellular level. Very recent studies have provided insights into the ‘hardware’ conduit of this connection between the CNS and the immune system with reports suggesting the involvement of the sympathetic nervous system (SNS). In animal experiments for example, evidence suggests that activating the reward system in mice modulates the immune system and that ablation of SNS nerves abolishes such modulation [57]. Such a conduit between CNS and immune function may also exist in human subjects. One study has shown that vagus nerve stimulation impacted cytokine production and attenuated the symptomatology of rheumatoid arthritis [58]. Other examples of such immune modulation include the effect of placebo Echinacea on the common cold where patients given placebo and active Echinacea had equal reduction in symptomatology and duration compared to those given no pills at all. For a subgroup of patients who believed in Echinacea and received pills, duration of colds was substantively shorter and less severe, regardless of whether the pills contained Echinacea [59].

Why might there have evolved an innate ability of parts of the CNS to use higher cognitive judgement of a situation the organism finds itself in to modulate seemingly automatic processes such as immune regulation? If one considers the considerable resources used in mounting and modulating immune activity [60], the ability of the CNS to judge the parsimonious use of such resources makes sense. This in the light of the need for differential immune responses in different situations where differing demands on resources are being encountered. For example, up regulating inflammatory responses are more useful in situations of social isolation whereas immune responses targeted at bacterial or viral infections are ramped down under such circumstances [61]. In addition, some circumstances including those that constitute a threat to safety would not be the most appropriate circumstances to rest, and recover or spend valuable resources on costly immune marshalling. Mechanisms that allow conscious complex social cues concerning present or near future circumstances of an individual to influence physiological responses such as immunity would then be evolutionarily advantageous. This is further explored in section 5.0.

### Motor function and performance

The influence of placebos in sport are only just beginning to be explored. Emerging evidence suggests that physical barriers to increased effort such as fatigue may be determined centrally in the CNS and not in the peripheral physiology. This leads to the idea that aspects of conscious expectation similar to those described in analgesia and immunity can also directly influence performance [62]. For example, deception of cyclists into believing they were racing against previous trials despite the trial demanding 2% greater power output than the actual previous trials, showed that such subjects always retained a metabolic reserve even during maximal time trials and that this reserve could be accessed after deception. Early studies involving placebo effects on muscle strength have shown that expectancy of receiving anabolic steroids significantly improved performance in weight lifters. Others have documented significant increases in power output in cyclists who believed they had ingested caffeine and that this was dose dependent [63]. Research then is emerging that documents significant and reproducible effects of placebo on performance and is linked to the ability to regulate pace, dependent upon CNS capacity, to cognitively predict the metabolic demands of exertion against actual metabolic capacity [64]. In this regard then, powerful cues similar to those seen in clinical practice may underlie such observed effects where a belief that improved performance will ensue post intervention.

## The false dichotomy of specific versus nonspecific

Nonspecific effects of treatment are characterised as those occurring incidentally outside the aim of the primary treatment and are interwoven with the negative semantics of the historical placebo. Placebo falls within the remit of such effects and the cultural impact of labelling treatment effects as nonspecific can be profound. The recent increasingly demonized practice of homeopathy for example has been dominated by the accusation that the clinical effects seen are nothing more than nonspecific in nature and as such, indefensible as a practice provided within the national healthcare system. Much of the evidence does indeed suggest that homeopathic approaches generate effects no greater than a placebo [65]. As another example, recent draft clinical guidelines for Low back pain in the UK have excluded acupuncture as a recommended modality precisely because the effects are non-specific in nature. To quote;

*“The GDG [Guideline Development Group] noted that although comparison of acupuncture with usual care demonstrated improvements in pain, function and quality of life in the short term, comparison with sham acupuncture showed no consistent clinically important effect, leading to the conclusion that the effects of acupuncture were probably the result of non-specific contextual effects* [66].

However, the crux of the matter (and often overlooked) is how much effect does a placebo generate? To speculate, if giving 100 patients a sugar pill in a context that generated adequate relief of IBS symptoms in 60% of patients, at a fraction of the cost of the standard pharmaceutical approach and with few side effects and near zero risk of harm, then arguments dismissing the effect as merely non-specific and worthless seems short sighted at the very least. Indeed, outside of speculation, similar results were seen with acupuncture augmented by positive language for IBS patients (63%) compared to a no treatment waiting list (28%) [67]. Why then a rejection of such phenomena in some quarters? Is it that acknowledgement of the efficacy of nonspecific effects threatens the nature of clinical authority and potentially in some eyes, undermines the cultural dominance of orthodoxy with fears of unfettered unorthodoxy? While such anxieties may be understandable to an extent, the wholesale dismissal of the overt use of such contextual factors as outlined in the CARe model might seem less than justified.

It is increasingly clear from emerging research that what is generating so called nonspecific effects is as specific as the binding of a drug to a receptor. This is exemplified by brain imaging studies that indicate that the well characterised pathways underlying expectation based analgesia are used in both the endogenous placebo response to pain and exogenous opioid drug response to pain and are substantively similar if not identical [68]. Indeed, evidence suggests that a range of drugs that elicit their ‘specific’ effects from analgesia to immune modulation do so by binding specifically to receptors on pre-existing neurological pathways that modulate the same activity that the drug is designed to modulate. In other words, much of the pharmacopeia specificity rides on the back of pre-existing pathways that can be modulated by contextual cues [69].

For contextually modulated analgesia, these neural pathways extend from the prefrontal cortex where the individual generates conscious understanding of context and meaning and end in the periaqueductal gray. Dominated by endorphin receptors, these networks, through further neural loci, modulate the ascending nociception signals generated by body tissue. Given these similarities in the neural pathways underlying both exogenous opioid drug based analgesia and endogenous endorphin based placebo analgesia the notion of specific and non specific based analgesia becomes meaningless. To illustrate, when an individual is given an opioid based drug, the drug *binds to receptors in an anatomically defined pain modulatory pathway and alters the activity of this pathway in a way that modulates the ascending nociceptive pain signals*. This is quintessential medicine, drug based, molecularly specific, chemically and biologically characterised action. When a clinician suggests that an inert placebo pill is a powerful analgesic, the patients understanding and expectation of pain relief triggered by these contextual signals releases ‘innate’ endogenous opioids (endorphins) that *binds to receptors in the same anatomically defined pain modulatory pathway as above and alters the activity of this pathway in a way that modulates the ascending nociceptive pain signals* [68]. This effect is called nonspecific, merely a placebo, and constitutes the basis for exclusion of a therapy if clinical trials indicate the efficacy is no better than placebo, despite that effect being large. Given that there is an iatrogenic epidemic of addiction to opioids in the US [70], yet few if any risks of addiction to ethically provided positive expectation as in CARe, how can this be justified? Furthermore, how can one effect be characterised as nonspecific and illegitimate and the other specific and legitimate, when they act through precisely the same biology using the same mechanism. This seems more like a cultural problem than a scientific one.

## The science of clinician mediated innate healing: why patients can’t do it alone

One key question that remains substantially unanswered within the study of contextual factors and their ability to modulate physiological processes centres on the apparent inability for an individual to ‘placebo’ themselves. Insights into an answer to this problem may provide a generic neurobiological basis of clinical interventions exemplified by the CARe paradigm presented here. This includes the skilful construction of contextual ‘wrappers’ or envelopes, within which degrees of specific modalities are embedded, forming the framework of skilled therapeutic and legitimate interventions.

To begin to explore answers to this question, it is important to consider a Darwinian context. Here the likely importance of the CNS to judge the use of physiological resources in the context of the circumstances the organism finds themselves, or anticipates to be in the near future is a key component. One area that may inform such a discussion is that of fatigue. Fatigue has been considered to be dominated by peripheral physiological factors, that ultimately result in failure of one or more systems, resulting in a physiological fatigue. However, a more recent formulation, the central governor model (CGM), invokes the idea of a central governor that through anticipatory psychological judgement, determines the pace needed to complete a physical task such as running, centrally generating a strong perception of fatigue if pace or distance is exceeded [70, 71].

Noakes [72] who originated this model suggests that factors integrated prior to a race to inform this predictive capacity may include “…*the athlete’s physiological state at the start of exercise; the expected distance or duration of the intended exercise bout; the degree of previous experience that the athlete has, especially in the specific activity that is being undertaken; the athlete’s level of motivation, which will be influenced by the level of external competition and the importance the athlete ascribes to the event; and the athlete’s level of self-belief*”.

Emerging evidence [73, 74] supports such a model, in the way athletes judge pacing as a complex interaction of emotions and expectations integrated with peripheral changes in muscles to support homeostasis during physical exercise. This makes evolutionary sense in that such an individual is protected from the effects of exhausting all resources while in an environment or an anticipated near future where additional resources may be called upon by some unpredictable event (e.g., encountering a lion at the end of running down an antelope precipitating the necessity of sprinting away) [75]. The idea of CNS judgement of circumstances within which resources can be appropriately spent, can be extend to answer the question why humans cannot self-generate strong placebo effects.

Nicholas Humphrey [75] first postulated the existence of a ‘health governor’ in 2002. This is similar but not identical to Noakes’ CGM where the CNS judgment of resource use is linked to fatigue [72]. In contrast, Humphrey envisaged a contextually driven process making top down predictions of the likely circumstances within which extensive resource use, such as those associated with increased marshalling of immune function [60], was likely to outweigh the downside of such resource use. Here the switching on of ‘innate healing processes’ were done only in the right conditions where other constraints such as food availability and environmental threats were likely to be minimised in the near future. This ancient ability would have initially been limited to coarse grained predictions at an unconscious level perhaps using light/dark changes to anticipate spring or winter [76].

Recently however, these authors and others [77, 78] have expanded this model to provide a comprehensive and convincing argument for the presence of additional input as provided by the evolution of higher cognitive function and social sharing of knowledge in humans. This system allows vastly improved predictions of the near future and could, in addition to the economic argument presented by these authors, be expanded to include the highly social nature of primates. This may help to explain why the contextual inclusion of powerful social figures such as clinicians, may be able to trigger hope of a ‘spring’ as opposed to a ‘winter’ and marshal self-healing.

In this model, one might envisage that the triggering of resources needed for recovery and rest are most *inappropriate* when a highly social animal like a primate is alone. In these circumstances vigilance and fight-flight are potentially more important with recovery being delayed or attenuated until cues indicate a degree of safety and protection are likely to be present and ongoing in the near future. For intensely social species such as homo sapiens these signals are likely to be most strongly conveyed by the presence of the family or kin group. For primates, cues such as touch through grooming may be additionally powerful signals of safety. Ultimately, grooming and or attentiveness by the alpha animal would be the strongest cues that the near future is likely to be one where spending expensive biological resources on recovery will result in more benefit than harm. In these circumstances it is envisaged that the CNS ‘health governor’ initiates a number of centrally determined physiological modulations, including pain, immune function and potentially others that switch the animal into recovery mode as opposed to survival. This idea posits grooming and attention as elements of prototypical medicine and socially elevated groomers as prototypical clinicians. Indeed, this may be why health professions taking on board the plumage of the culturally dominant clinical paradigm (titles, clothing, language, context) elicit powerful contextual triggers that generate innate modulation of health states.

**Fig. 1:**
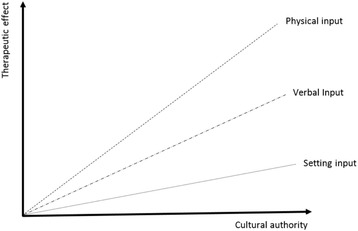
Three component model of contextual factors modulated by cultural authority

The predictions of such a model are intriguing. For example, one might suggest that there are three broad contextual components that may interact: physical signals (touch being prototypical), verbal cues, and environmental cues. In addition, the impact of these factors may be modulated by the degree of cultural authority that the individual is perceived as possessing and this may be signalled by the cultural attributes that serve to engender authority and trust [79]. This may be summarised in Fig. 1.

**Fig. 2:**
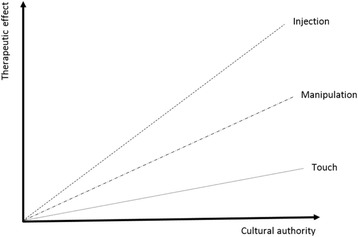
Increased physical intervention enhances contextual effects

In addition, increasing physical invasiveness (either perceived or real) has increased impact on modulation of the contextual effect (for example analgesia). Speculating in the arena of manual therapy, touch through manipulation to injection may be having a more powerful effect of pain modulation within the correct delivery of the other contextual domains (Fig. 2). In support of such a conjecture such grading of effects as generated by contextual factors has previously been documented [80–82]. For example, needle acupuncture, has been shown to be more effective than other less invasive procedures [82]. Furthermore, combined chiropractic interventions have been shown to be slightly better at improving acute low back pain and disability in the short term, and pain in the medium term, than less “invasive” modalities such as massage or stretching exercises [83].

This model predicts a number of testable research questions.

1. In the context of manual therapy, do soft tissue interventions alone have smaller effect sizes in low back pain patients than those seen in manipulation and facet injection?
2. Furthermore, do casually dressed and uncertain chiropractors generate smaller analgesic effects than those in a white coat and/or proclaiming certainty about the efficacy of the treatment?
3. Does delivery of care in a clinically designed aesthetic chiropractic setting generate better analgesic effects than those delivered in the patient’s own home.
4. Does a comparison of strongly positive verbal cueing as compared to neutral verbal cueing produce increased benefit in chiropractic settings?

This model also suggests that there may be combinations of contextual factors within these domains that are more or less effective. For example, it is already known that verbal cues can modulate the effect of both drug and placebo effectiveness in reducing the severity of migraine [80] and in modulating analgesia in acupuncture [84]. Lastly, there is a possibility that different subgroups of patients may be more or less amenable to various combinations of contextual factors including the timing of such factors during the course of a condition. Indeed, evidence is already emerging regarding genetic predispositions to contextually induced analgesia [85].

Taken together, the CARe model and the preceding summary of research provides an argument for an approach to clinical care that aims to maximise through detailed understanding of combinations of contextual factors and targeting of patients, the recovery or management of a range of conditions in addition to those associated with musculoskeletal (MSK) pain. Knowledgeable and judicious use of these factors in the construction of a therapeutic structure can generate powerful cues that support an interpretation by the patients ‘health governor’ that safety and care will be available in the predicted near future. Within these environments, intrinsic recuperative mechanisms including pain and immune modulation can be switched on by anthropologically and evolutionary informed environmental, verbal and physical signals as delivered in a cultural context. These processes are in turn controlled by CNS based networks/pathways that are integrated with higher cognitive and learned processes that anticipate appropriate use of biological resourcing. Skilful delivery of such care, being as it is associated with recovery, should then be couched in a language far removed from the traditional polemic of placebo.

CARe as described here could in the future constitute a shift in the false dichotomy between specific and nonspecific therapeutic effects and the policy implications therein underpinned as they are by neurobiological and physiological science. Such an understanding may provide the foundations of increasing acceptance of these skills and the effects they generate as legitimate therapeutic intervention in addition to others, or in and of itself. A word of caution. Ethical issues remain [13, 86] regarding deception and the fact that placebos rarely cure, although a recent study indicated open placebo without deception to provide additional effectiveness over and above normal care in attenuating symptoms in low back pain patients [87]. Notwithstanding, clear boundaries would be imperative with a focusing of such care on ostensibly non-life threatening conditions or the sequelae of other conditions such as chronic pain [88]. In addition, embracing such a different view of what legitimate treatment constitutes, may be a challenge not only in some of the manual therapeutic professions but also to medicine itself. In the end, however, the welfare of the patient and best practice as increasingly and rightly centred on the needs and experience of patients may allow a re-evaluation of how legitimate clinical care is described and in what capacity it is best delivered.

## Conclusion

This paper introduces CARe as a new descriptive model of the power of contextual factors and their effects on analgesia, immune and motor modulation in MSK conditions.

Evidence has mounted over the last decade to support the ability of the CNS to judge the context of a clinical encounter, what such contextual cues imply about the predicted near future, biological resource use and the impact of such judgements on physiological processes through expectation and classical conditioning mechanisms. This emerging understanding could provide a scientific basis for a proportion of the observed MSK effects and much of non-MSK effects seen in chiropractic patients undergoing chiropractic care. Indeed, a recent study comparing an effective sham [89] with spinal manipulation as guided by Gonstead methodology in migraine patients concluded that the positive effects in the manipulation group were the same as in the sham group, both groups better than the control group, and were likely due to a placebo response [90].

Professional anxieties understandably exist and the pejorative language of placebo is an unhelpful and inaccurate way to describe and judge these effects creating as they do a viewpoint that relegates such skilled construction of therapeutic encounters to no more than subterfuge or fraud. We present a contrasting view that suggest these powerful and clinically important effects should be further investigated, maximised and embraced by all healthcare professions where the nature of patient complaints is most benefited by such contextually laden care, particularly chronic pain [89]. In addition, the need for a caring, highly knowledgeable and skilful clinician to enable individuals to access these innate healing systems suggests an increasing imperative to embed aspects of models such as CARe in clinical encounters and clinical education while elucidating more clearly the therapeutic components through clinical research.

The CARe approach should be an essential and powerful therapeutic tool that is central to the chiropractic clinical encounter and may provide a framework for the profession to engage in scientific investigation and debate around such effects and in so doing relinquish unnecessary adherence to outmoded and non-evidenced paradigms as explanations for observations made in clinical practice.

Much research still needs to be carried out to begin to uncover the precise nature of contextual factors and their interrelationships within the typical chiropractic encounter. Nevertheless, it is hoped that the CARe paradigm may provide an evidence based framework for legitimising skilful and patient centred application of contextual healing within the chiropractic profession and across health care in general.

## Abbreviations

CARe: Contextually Aided Recovery
CGM: Central governor model
CNS: Central nervous system
GDG: Guideline development group
MSK: Musculoskeletal
SNS: Sympathetic nervous system
UK: United Kingdom
